# Supplementary material for: Comparative Analysis of L-Fucose Utilization and Its Impact on Growth and Survival of Campylobacter Isolates
Source: Front Microbiol. 2022 Apr 29;13:872207. doi: 10.3389/fmicb.2022.872207 (PMC9100392; doi:10.3389/fmicb.2022.872207)
Supplement: Supplementary file 1 [file Data_Sheet_1.docx]

**Supplementary**

**Table. S1. Components of 41061 - MEM alpha, nucleosides, no phenol red**

| Components | mM | Components | mM |
| --- | --- | --- | --- |
| **Amino Acids** |  | **Inorganic Salts** |  |
| Glycine | 0.67 | Calcium Chloride (CaCl_2_) (anhyd.) | 1.80 |
| L-Alanine | 0.28 | Magnesium Sulfate (MgSO_4_) (anhyd.) | 0.81 |
| L-Arginine | 0.49 | Potassium Chloride (KCl) | 5.33 |
| L-Asparagine-H_2_O | 0.33 | Sodium Bicarbonate (NaHCO_3_) | 26.19 |
| L-Aspartic acid | 0.23 | Sodium Chloride (NaCl) | 117.24 |
| L-Cysteine hydrochloride-H_2_O | 0.57 | Sodium Phosphate monobasic (NaH_2_PO_4_-H_2_O) | 1.01 |
| L-Cystine 2HCl | 0.10 | **Ribonucleosides** |  |
| L-Glutamic Acid | 0.51 | Adenosine | 0.037 |
| L-Glutamine | 2.0 | Cytidine | 0.041 |
| L-Histidine | 0.2 | Guanosine | 0.035 |
| L-Isoleucine | 0.4 | Uridine | 0.040 |
| L-Leucine | 0.40 | **Deoxyribonucleosides** |  |
| L-Lysine | 0.40 | 2'Deoxyadenosine | 0.039 |
| L-Methionine | 0.10 | 2'Deoxycytidine HCl | 0.042 |
| L-Phenylalanine | 0.19 | 2'Deoxyguanosine | 0.037 |
| L-Proline | 0.35 | Thymidine | 0.041 |
| L-Serine | 0.24 | **Other Components** |  |
| L-Threonine | 0.40 | D-Glucose (Dextrose) | 5.55 |
| L-Tryptophan | 0.05 | Lipoic Acid | 9.71E-04 |
| L-Tyrosine disodium salt | 0.23 | Sodium Pyruvate | 1.00 |
| L-Valine | 0.39 |  |  |
| **Vitamins** |  |  |  |
| Ascorbic Acid | 0.28 |  |  |
| Biotin | 4.10E-04 |  |  |
| Choline chloride | 0.01 |  |  |
| D-Calcium pantothenate | 0.002 |  |  |
| Folic Acid | 0.002 |  |  |
| Niacinamide | 0.008 |  |  |
| Pyridoxal hydrochloride | 0.005 |  |  |
| Riboflavin | 2.66E-04 |  |  |
| Thiamine hydrochloride | 0.003 |  |  |
| Vitamin B12 | 0.001 |  |  |
| i-Inositol | 0.01 |  |  |

**Table. S2. Overview of genes in the L-fucose utilization cluster**

**Fig. S1. Microscopic images of the tested *Campylobacter* isolates**


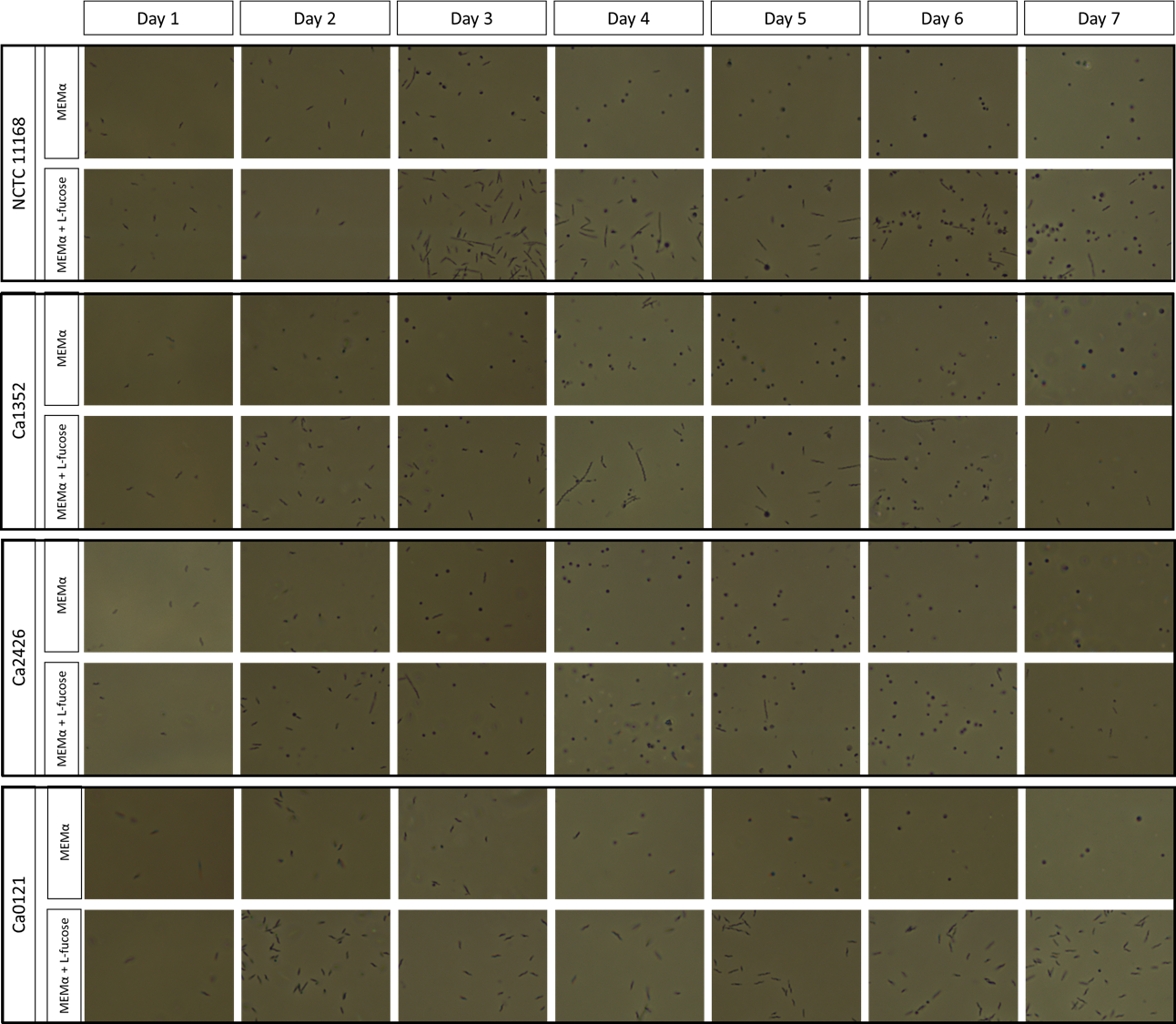


Figure S1 Morphology of C. jejuni NCTC11168, Ca1352, Ca2426 and C. coli Ca0121 during a 7 day growth experiment in MEMα or MEMαF medium.

**Fig. S2. Growth and L-fucose consumption of *C. jejuni* NCTC11168, Ca2426 and *C. coli* Ca0121 in MH2 and MH3 medium**


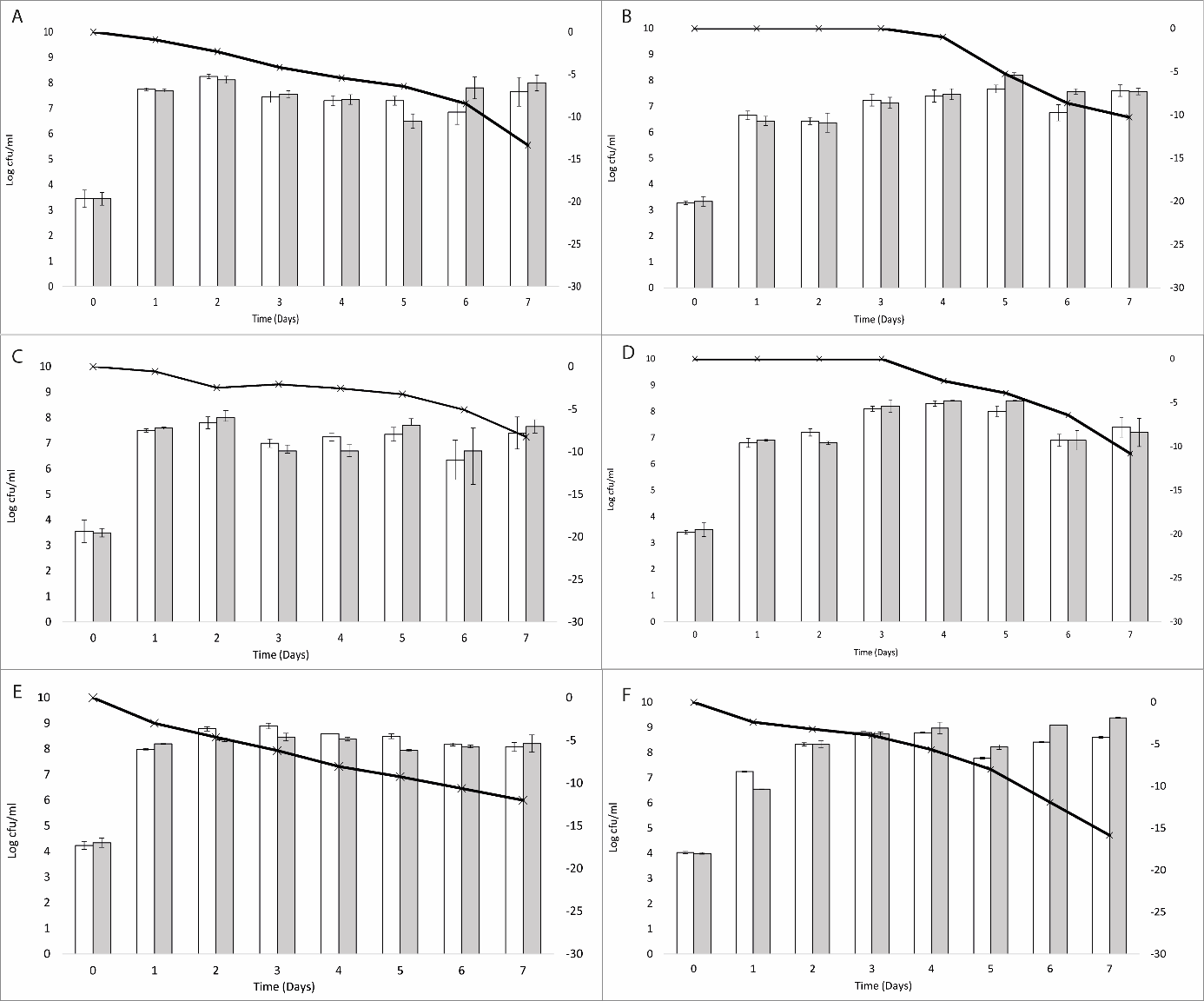


Figure S2. Quantification of planktonic growth for **A)** isolate C. jejuni NCTC11168 in MH2 medium , **B)** C. jejuni NCTC11168 in MH3 medium **C)** C. jejuni Ca2426 in MH2 medium, **D)** C. jejuni Ca2426 in MH2 medium, **E)** C. coli Ca0121 in MH2 medium and **F)** C. coli Ca0121 in MH3 medium. White bars represent MH2 or MH3 medium and grey bars represent MH2 or MH3 medium + L-fucose. The black line shows consumption of L-fucose over time.

**Fig. S3. HPLC measurements of amino acids**

**
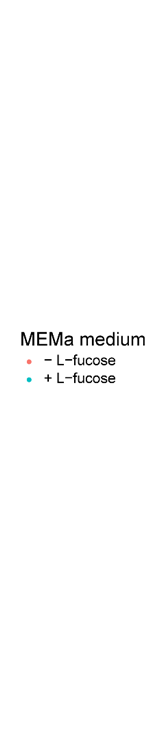
**
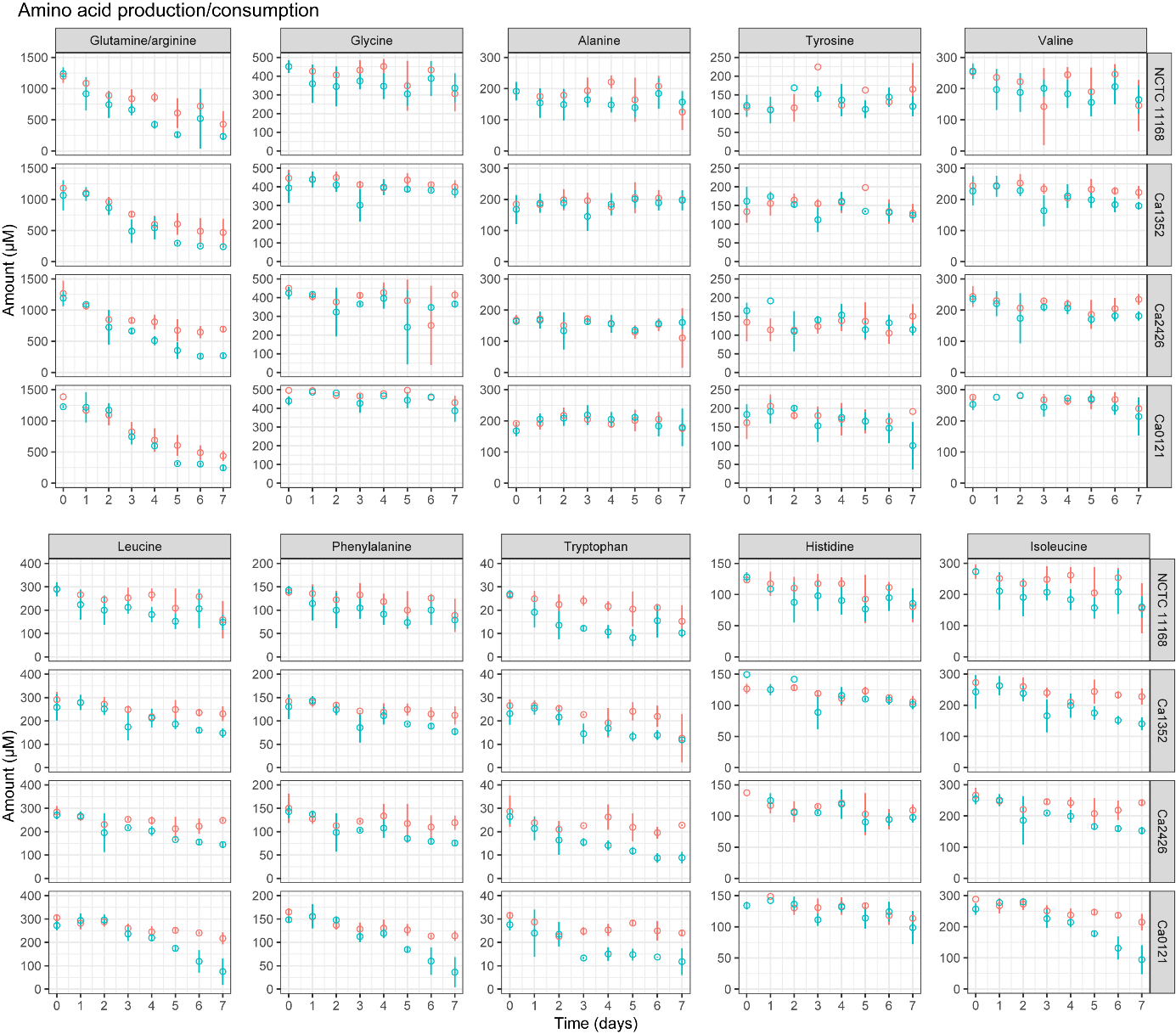


Figure 2 Amino acid production and consumption of selected Campylobacter isolates in MEMαF medium (blue dots) and MEMα medium (red dots) after 7-day incubation period. Columns correspond to different amino acids and each row shows results for a different isolate. Each value represents the average of three biologically independent replicates, and error bars show the standard deviation.

**Fig. S4. Complete L-fucose gene cluster alignment**

***Cj0480C***

NCTC11168 1 MHQPTLRVLNILELLAKEKLTLSAIAKKLNIPAGTLWPILQTLQEKKYIKCDLKNKSYYL
Ca1352 1 MHQPTLRVLNILELLAKEKLTLSAIAKKLNIPAGTLWPILQTLQEKKYIKCDLKNKSYYL
2426 1 MHQPTLRVLNILELLAKEKLTLSAIAKKLNIPAGTLWPILQTLQEKKYIKCDLKNKSYYL
Ca0121 1 MHQPTLRVLNILELLAKEKLTLSTIAKKLNIPAGTLWPILQTLQEKQYIRCDLKNKSYYL

NCTC11168 61 DFKIIELGCNIKNENNIFEIIKKHMKNIRNLTNQTCQMGILKDGNVLYLEKIDANNTVQL
Ca1352 61 DFKIIELGCNIKNENNIFEIIKKHMKNIRNLTNQTCQMGILKDGNVLYLEKIDANNTVQL
2426 61 DFKIIELGCNIKNENNIFEIIKKHMKNIRNLTNQTCQMGILKDGNVLYLEKIDANNTVQL
Ca0121 61 DFKILELGYNIKSENSIFEMIKKHMKNIRNLTNQTCQMGILKEENVLYLEKIDANNPVQL

NCTC11168 121 KSFIGTSYPAYATSLGKALLSNKNKKELEKLYPKNFDKITENTLNNINELYQQIKQIKKE
Ca1352 121 KSFIGTSYPAYATSLGKALLSNKNKKELEKLYPKNFDKITENTLNNINELYQQIKQIKKE
2426 121 KSFIGTSYPAYATSLGKALLSNKNKKELEKLYPKNFDKITENTLNNINELYQQIKQIKKE
Ca0121 121 KSFIGTSYPAYATSLGKALLSNKNKEELKKIYPNNFEKITENTLKNIDELYQQIEQIKKE

NCTC11168 181 KIAIEIGEMNPQIECMAIGIEHKNKIIAAISISYLIYCSNKAFREKNKKILLEEKNKIEK
Ca1352 181 KIAIEIGEMNPQIECMAIGIEHKNKIIAAISISYLIYCSNKAFREKNKKILLEEKNKIEK
2426 181 KIAIEIGEMNPQIECMAIGIEHKNKIIAAISISYLIYCSNKAFREKNKKILLEEKNKIEK
Ca0121 181 KIAIESGEINPQIECMAIGIEHKNKIIAAISISYLIFYSNETFREKNKKILLEEKNKIEK

NCTC11168 241 VLKIYFNDLDTLY
Ca1352 241 VLKIYFNDLDTLY
2426 241 VLKIYFNDLDTLY
Ca0121 241 ALKIHFNDLDTLY

***Cj0481***

NCTC11168 1 MGILKGTLPALLTPYKDDGSINEKEFIRYCEFGISKGLNGLFCNGSAGDSQALSVEEQVK
Ca1352 1 MGILKGTLPALLTPYKDDGSINEKEFIRYCEFGISKGLNGLFCNGSAGDSQALSVEEQVK
Ca2426 1 MGILKGTLPALLTPYKDDGSINEKEFIRYCEFGISKGLNGLFCNGSAGDSQALSVEEQVK
Ca0121 1 MGILKGTLPALLTPYKDDGSINEKEFIHYCEFGISKGLNGLFCNGSAGDSQALSIEEQVK

NCTC11168 61 LMKLTKKATKNNIPIITGITSTIYENTFILAQKAYELGLDALLLAMPYYYKLSEDALFEY
Ca1352 61 LMKLTKKATKNNIPIITGITSTIYENTFILAQKAYELGLDALLLAMPYYYKLSEDALFEY
Ca2426 61 LMKLTKKATKNNIPIITGITSTIYENTFILAQKAYELGLDALLLAMPYYYKLSEDALFEY
Ca0121 61 LMRLTKEVAKNNVPIITGIASTIYQNTFTLAQKAYEIGLDALLLAMPYYYKLSEDALFEY

NCTC11168 121 VKYLASEVKLPLYVYNIPLFAPALSLKFIEKVSKLDNVVGIKDSSGDALLLNHILDVVPS
Ca1352 121 VKYLASEVKLPLYVYNIPLFAPALSLKFIEKVSKLDNVVGIKDSSGDALLLNHILDVVPS
Ca2426 121 VKYLASEVKLPLYVYNIPLFAPALSLKFIEKVSKLDNVVGIKDSSGDALLLNHILDVVPS
Ca0121 121 VKDLSSKVKLPLYVYNIPLFAPALSLKFIERVSKLDNVVGIKDSSGDALLLNHILDVLPS

NCTC11168 181 NFDVFVGREEFYVGALLAGVKGSMTSIGGVFPELMSEIYKSINEKNIGRALLIQKSLLKA
Ca1352 181 NFDVFVGREEFYVGALLAGVKGSMTSIGGVFPELMSEIYKSINEKNIGRALLIQKSLLKA
Ca2426 181 NFDVFVGREEFYVGALLAGVKGSMTSIGGVFPELMSEIYKSINEKNIGRALLIQKSLLKA
Ca0121 181 NFDVFVGREEFYVGALLAGAKGSMTSIGGVFPELMSEIYKSINEKNIGRALLIQKSLLKA


NCTC11168 241 IRFGMSIAFPMGFALLLKARGFEFVNASIHPLSPATKEELNTRFDEAKELIKTIEKETGI
Ca1352 241 IRFGMSIAFPMGFALLLKARGFEFVNASIHPLSPATKEELNTRFDEAKELIKTIEKETGI
Ca2426 241 IRFGMSIAFPMGFALLLKARGFEFVNASIHPLSPATKEELNTRFDEAKELIKTIEKETGI
Ca0121 241 IRFGMSIAFPMGFALLLKARGFEFVNASIHPLSPATKEELNTRFDEAKELIKTIEKETGI

NCTC11168 301 KL
Ca1352 301 KL
Ca2426 301 KL
Ca0121 301 KL

***Cj0482***

NCTC11168 1 MKGYIIVNEKDNVATALRDFKKGEKVANIELLNDIASGHKFALKDIKKDEIIIKYAEAIA
Ca1352 1 MKGYIIVNEKDNVATALRDFKKGEKVANIELLNDIASGHKFALKDIKKDEIIIKYAEAIA
Ca2426 1 MKGYIIVNEKDNVATALRDFKKGEKVANIELLNDIASGHKFALKDIKKDEIIIKYAEAIA
Ca0121 1 MKGYIIVNEKDNVATALRDFKKGEKVANIELLNDIASGHKFALKDIKKDEIIIKYAEAIA

NCTC11168 61 SASCDISTGEWVHIHNTAGIRGRGDKE
Ca1352 61 SASCDISTGEWVHIHNTAGIRGRGDKE
Ca2426 61 SASCDISTGEWVHIHNTAGIRGRGDKE
Ca0121 61 SASCDISTGEWVHIHNTAGIRGRGDKE

***Cj0483***

NCTC11168 1 MKKIMGYRREDGKFGLRNKVIIIPSVHCANKVCENIARKCNGAVYINHQHGCSQLEFDAL
Ca1352 1 MKKIMGYRREDGKFGLRNKVIIIPSVHCANKVCENIARKCNGAVYINHQHGCSQLEFDAL
Ca2426 1 MKKIMGYRREDGKFGLRNKVIIIPSVHCANKVCENIARKCNGAVYINHQHGCSQLEFDAL
Ca0121 1 MKKIMGYRREDGKFGLRNKVIIIPSVHCANKVCENIARKCNGAVYINHQHGCSQLEFDAL

NCTC11168 61 QTRDVLIGHGSNANVFGVLIIGLGCEVIQAKAVAEKIKEAMPYKKVEYLVIQECGGSKNT
Ca1352 61 QTRDVLIGHGSNANVFGVLIIGLGCEVIQAKAVAEKIKEATPYKKVEYLVIQECGGSKNT
Ca2426 61 QTRDVLIGHGSNANVFGVLIIGLGCEVIQAKAVAEKIKEATPYKKVEYLVIQECGGSKNT
Ca0121 61 QTRDVLIGHGSNANVFGVLIVGLGCEVIQAKVVAEKIKEATPYKKVEYLVIQECGGSKNT

NCTC11168 121 IENGIKIVNEMLESAAKLQKSEGDFSDLILGTECGGSDSYSGLSANPTLGSLSDFVIDEG
Ca1352 121 IENGIKIVNEMLESAAKLQKSEGDFSDLILGTECGGSDSYSGLSANPALGSLSDFVIDEG
Ca2426 121 IENGIKIVNEMLESAAKLQKSEGDFSDLILGTECGGSDSYSGLSANPALGSLSDFVIDEG
Ca0121 121 IEKGIKIVNEMLESAAKLQKSEGDFSDLILGTECGGSDSYSGLSANPALGSLSDFVIDEG

NCTC11168 181 GAVILAETTELIGCEAILAKRAKNDEIAKKVYDKILAYENLVKSFHADIRGANPSPGNMA
Ca1352 181 GAVILAETTELIGCEAILAKRAKNDEIAKKVYDKILAYENLVKSFHADIRGANPSPGNMA
Ca2426 181 GAVILAETTELIGCEAILAKRAKNDEIAKKVYDKILAYENLVKSFHADIRGANPSPGNMA
Ca0121 181 GAVILAETTELIGCEAILAKRAKNDEIAKKVYDKILGYENLVKSFRADIRGANPSPGNIA

NCTC11168 241 GGLSTIEEKSLGCVYKAGTRTLMDVIDYAKPVVSKGLTFMNTPGNDIEQLSAMVAGGANI
Ca1352 241 GGLSTIEEKSLGCVYKAGTKTLMDVIDYAKPVVSKGLTFMNTPGNDIEQLSAMVAGGANI
Ca2426 241 GGLSTIEEKSLGCVYKAGTKTLMDVIDYAKPVVSKGLTFMNTPGNDIEQLSAMVAGGANI
Ca0121 241 GGLSTIEEKSLGCVYKAGTRTLMDVIDYAKPIVSKGLTFMNTPGNDIEQLSAMVAGGANI
NCTC11168 301 CVFTTGRGTPTGSAIVPTIKMSSNTFCYENMNDAIDINAGSIIDGVKTKEEVRDELIELI
Ca1352 301 CVFTTGRGTPTGSAIVPTIKMSSNTFCYENMNDAIDINAGSIIDGVKTKEEVRDELIELI
Ca2426 301 CVFTTGRGTPTGSAIVPTIKMSSNTFCYENMNDAIDINAGSIIDGVKTKEEVRDELIELI
Ca0121 301 CVFTTGRGTPTGSAIVPTIKMSSNTFCYENMNDAIDINAGSIIDGVKTKEEVRDELIELI

NCTC11168 361 VRISDGELVKAELNEQNDFSAWRLATTC
Ca1352 361 VRISDGELVKAELNEQNDFSVWRLATTC
Ca2426 361 VRISDGELVKAELNEQNDFSVWRLATTC
Ca0121 361 VRISDGELVKAEINEQNDFSVWRLATTC

***Cj0484***

NCTC11168 1 MKHANSIKLELVCKKISWRILPLIVLMFCLSMLDRTNISFVKSHIEIDAGIGEAAYALGA
Ca1352 1 MKHANSIKLELVCKKISWRILPLIVLMFCLSMLDRTNISFVKSHIEIDAGIGEAAYALGA
Ca2426 1 MKHANSIKLELVCKKISWRILPLIVLMFCLSMLDRTNISFVKSHIEIDAGIGEAAYALGA
Ca0121 1 MGHLKLTKLELVCKKISWRILPLIVLMFCLSMLDRTNISFVKSHIEMDAGISETAYALGA

NCTC11168 61 GIFFIGYAIFEVPSNLFLHKLGAKIWLSRIMITWGLVTMAMIFIQGEISFYVLRFLLGLT
Ca1352 61 GIFFIGYAIFEVPSNLFLHKLGAKIWLSRIMITWGLVTMAMIFIQGEISFYVLRFLLGLT
Ca2426 61 GIFFIGYAIFEVPSNLFLHKLGAKIWLSRIMITWGLVTMAMIFIQGEISFYVLRFLLGLT
Ca0121 61 GIFFVGYALFEVPSNLLLHKLGAKIWLSRIMITWGLVTMAMIFIQGETSFYILRFFLGLT

NCTC11168 121 EAGFSPGIILYLSYFFPAIYRSKAYGIYQMGVPIAFVFGSLISGFILDYTPNIYFKNWQW
Ca1352 121 EAGFSPGIILYLGYFFPAIYRSKAYGIYQMGVPIAFVFGSLISGFILDYTPNIYFKNWQW
Ca2426 121 EAGFSPGIILYLGYFFPAIYRSKAYGIYQMGVPIAFVFGSLISGFILDYTPNIYFKNWQW
Ca0121 121 EAGFSPGIILYLSYFFPAIYRSKAYGIYQMGVPIAFVFGSLISGFILDYASNMYFKNWQW

NCTC11168 181 MFLIEGGITVLVGIFCLFYLDSHPKDAKWLDIKEKDILLKHIEISNTKAKDYSIKDIFKS
Ca1352 181 MFLIEGGITVLVGIFCLFYLDSHPKDAKWLDIKEKDILLKHIEISNTKAKDYSIKDIFKS
Ca2426 181 MFLIEGGITVLVGIFCLFYLDSHPKDAKWLDIKEKDILLKHIEISNTKAKDYSIKDIFKS
Ca0121 181 MFLIEGGITVLVGVFCLFYLDSHPKDAKWLDVKEKTVLLKNIEICNSKAKDYSIKDIFKN

NCTC11168 241 ILVWKFVFVYFCIQLSVYGVLFYLPSKIAQILQINVGFEVGLLNAIPWIFVFIALPIFTS
Ca1352 241 ILVWKFVFVYFCIQLSVYGVLFYLPSKIAQILQINVGFEVGLLNAIPWIFVFIALPIFTS
Ca2426 241 ILVWKFVFVYFCIQLSVYGVLFYLPSKIAQILQINVGFEVGLLNAIPWIFVFIALPIFTS
Ca0121 241 ILVWKFVFVYFCIQLSVYGVLFYLPSKIAQILQINVGFEVGLLNAIPWIFVFIALPIFTS

NCTC11168 301 LADKKHSWNLHAILFLLLASLSMIASTFVTNLAAFLFFISLAAIGFIVIQPIFWNLPTQV
Ca1352 301 LADKKHSWNLHAILFLLLASLSMIASTFVTNLAAFLFFISLAAIGFIVIQPIFWNLPTQV
Ca2426 301 LADKKHSWNLHAILFLLLASLSMIASTFVTNLAAFLFFISLAAIGFIVIQPIFWNLPTQV
Ca0121 301 LADKKRSWNLYAILFLLLASLSMIASTFVANLAPFLFFISLAVIGFIVIQPIFWNLPTQV

NCTC11168 361 LKGKGAAAAIALIGSLGNLGGFVAPTLKTYIENHFGVEFGLIVLALIAIL----------
Ca1352 361 LKGKGAAAAIALIGSLGNLGGFVAPTLKTYIENHFGVEFGLIVLALIAILGVLVLIHLKI
Ca2426 361 LKGKGAAAAIALIGSLGNLGGFVAPTLKTYIENHFGVEFGLIVLALIAILGVLVLIHLKI
Ca0121 361 LKGKGGAAAIALIGSLGNLGGFVAPTLKTYIENHFGVEFGLITLALIAVLGVLILVHLKM

NCTC11168 --------
Ca1352 421 TLNLDKGE
Ca2426 421 TLNLDKGE
Ca0121 421 TLN-----

***Cj0485***

NCTC11168 1 MDLKIKNKVCIITGGAKGIGYGIAKLWASEGGIPVIFSRSMPKEHDKELKKLSSEYEFYE
Ca1352 1 MDLKIKNKVCIITGGAKGIGYGIAKLWASEGGIPVIFSRSMPKEHDKELKKLSSEYEFYE
Ca2426 1 MDLKIKNKVCIITGGAKGIGYGIAKLWASEGGIPVIFSRSMPKEHDKELKKLSSEYEFYE
Ca0121 1 MDLKIKNKVCIITGGAKGIGYGIAKLWAMEGGIPVVFSRSMPKEHDEELKNLCKSYEFYE

NCTC11168 61 IDLKNYEQIEKLVKKVAIKHGGIYALVNNAGTNDNLHIENTSTQDLIKSYENNLFHYYTM
Ca1352 61 IDLKNYEQIEKLVKKVAIKHGGIYALVNNAGTNDNLHIENTSTQDLIKSYENNLFHYYTM
Ca2426 61 IDLKNYEQIEKLVKKVAIKHGGIYALVNNAGTNDNLHIENTSTQDLIKSYENNLFHYYTM
Ca0121 61 IDLKNYEQIEELIKKVVTKHGGIYALVNNAGANDNLHIENTSTKDLIKSYENNLFHYYAM

NCTC11168 121 TKECLPYIKKEQGSILNIVSKTGITGQGRTSAYASAKAAQMGFTREWACAFAKDNVRVNA
Ca1352 121 TKECLPYIKKEQGSILNIVSKTGITGQGRTSAYASAKAAQMGFTREWACAFAKDNVRVNA
Ca2426 121 TKECLPYIKKEQGSILNIVSKTGITGQGRTSAYASAKAAQMGFTREWACAFAKDNVRVNA
Ca0121 121 AKECLPYIKKEQGSILNIVSKTGITGQGRTSAYASAKAAQIGFTREWACAFAKDSVRVNA

NCTC11168 181 IAPAEVMTPLYEKWLQNFPNPKEQYEKIAKAIPLGHRFTTIEEIANTAVFTLSPLASHTT
Ca1352 181 IAPAEVMTPLYEKWLQNFPNPKEQYEKIAKAIPLGHRFTTIEEIANTAVFTLSPLASHTT
Ca2426 181 IAPAEVMTPLYEKWLQNFPNPKEQYEKIAKAIPLGHRFTTIEEIANTAVFTLSPLASHTT
Ca0121 181 IAPAEVMTPLYEKWLQNFPNPKEQYEKIAKTIPLGHRFTTIEEIANTAVFTLSPLASHTT

NCTC11168 241 GQILMPDGGYVHLDRALNWDEN
Ca1352 241 GQILMPDGGYVHLDRALNWDEN
Ca2426 241 GQILMPDGGYVHLDRALNWDEN
Ca0121 241 GQILMPDGGYVHLDRALNWDEI

***Cj0486***

NCTC11168 1 MTDSKNIKIAIVLVTSLFFLWGVSYGLIDVMNKNFQNHLHISQHESGFLQFAYFGAYFII
Ca1352 1 MTDSKNIKIAIVLVTSLFFLWGVSYGLIDVMNKNFQNHLHISQHESGFLQFAYFGAYFII
Ca2426 1 MTDSKNIKIAIVLVTSLFFLWGVSYGLIDVMNKNFQNHLHISQHESGFLQFAYFGAYFII
Ca0121 1 MTDSKNIKIAIVLVTSLFFLWGVSYGLIDVMNKNFQNHLHISQHESGFLQFAYFGAYFII

NCTC11168 61 ALPAGYIANRFSYKMGIIFGLALYAIGALLIIPATNLASFHLFLFAFFILACGIGSLETS
Ca1352 61 ALPAGYIANRFSYKMGIIFGLALYAIGALLIIPATNLASFHLFLFAFFILACGIGSLETS
Ca2426 61 ALPAGYIANRFSYKMGIIFGLALYAIGALLIIPATNLASFHLFLFAFFILACGIGSLETS
Ca0121 61 ALPAGYIANKYSYKMGIIFGLALYAVGALLIIPATNLASFHLFLFAFFVLACGIGSLETS

NCTC11168 121 ANPYMTKLGDEKNASFRINAAQSFNGLGQFVGPIIGGALFLSITKQEEGASKEQIQAALV
Ca1352 121 ANPYMTKLGDEKNASFRINAAQSFNGLGQFVGPIIGGALFLSITKQEEGASKEQIEAALV
Ca2426 121 ANPYMTKLGDEKNASFRINAAQSFNGLGQFVGPIIGGALFLSITKQEEGASKEQIEAALV
Ca0121 121 ANPYMVKLGDEKNASFRINAAQSFNGLGQFVGPIIGGALFLSITKQEEGASAEQIEAALL

NCTC11168 181 ANMGNVQLVYIGIAVIVILILIAFVANKLPEGSAVSDDYKQKDDSKPIYVFKHRHFNLGL
Ca1352 181 ANMGNVQLVYIGIAVIVILILIAFVANKLPEGSAVSDDYKQKDDSKPIYVFKHRHFNLGL
Ca2426 181 ANMGNVQLVYIGIAVIVILILIAFVANKLPEGSAVSDDYKQKDDSKPIYVFKHRHFNLGL
Ca0121 181 ANMGNVQLVYVGIAAIVILILIAFVLNKLPEGSAVSDDYKQKDDSKPIHVFKHRHFNLGL

NCTC11168 241 LAQFLYIANQVAAGAFFINYVVEHNEGLKDAQGAYYFSIALVAFMLGRIVSTPLMKIIKG
Ca1352 241 LAQFLYIANQVAAGAFFINYVVEHNEGLKDAQGAYYFSIALIAFMLGRIVSTPLMKIIKG
Ca2426 241 LAQFLYIANQVAAGAFFINYVVEHNEGLKDAQGAYYFSIALIAFMLGRIVSTPLMKIIKG
Ca0121 241 LAQFSYIANQVAAGAFFINYVVDHNEGLKDAQAAYYFSIALVAFMLGRIVSTPLMKVIKG

NCTC11168 301 EKILGFYSLINVLICFSLYFASGFFSIVLLIALFFFMSISFPTIFAVATKNLPLNQVKLG
Ca1352 301 EKILGFYSLINVLICFSLYFASGFFSIVLLIALFFFMSISFPTIFAVATKNLPLNQVKLG
Ca2426 301 EKILGFYSLINVLICFSLYFASGFFSIVLLIALFFFMSISFPTIFAVATKNLPLNQVKLG
Ca0121 301 EKILGLYSLINVLICFGLYFTSGFLSTILLIALFFFMSISFPTIFAVATKNLPLNQVKLG

NCTC11168 361 GSLLVMSIVGGAIMPIIIGFINDHYGTGAGYLAMAPLFLYVAWYGFIGSKVRKNAKDF
Ca1352 361 GSLLVMSIVGGAIMPIIIGFINDHYGTGAGYLAMAPLFLYVAWYGFIGSKVRKNAKDF
Ca2426 361 GSLLVMSIVGGAIMPIIIGFINDHYGTGAGYLAMAPLFLYVAWYGFIGSKVRKNAKDF
Ca0121 361 GSLLVMSIVGGAIMPLIIGFINDHYGTGMGYLAMAPLFLYVAWYGFVGSKIKA-----

***Cj0487***

NCTC11168 1 MQKIFDAHLHLWDLEKIPISWIKDDEKLEQNYDFFRMKQEYKEFEFIGAMYVEVNSDDLE
Ca1352 1 MQKIFDAHLHLWDLEKIPISWIKDDEKLEQNYDFFRMKQEYKEFEFIGAMYVEVNSDDLE
Ca2426 1 MQKIFDAHLHLWDLEKIPISWIKDDEKLEQNYDFFRMKQEYKEFEFIGAMYVEVNSDDLE
Ca0121 1 MQKIFDAHLHLWDLDKMPISWLKGNEKLEQNYDFFRAKEEYEGFEFLGAMYVETNSDDLE

NCTC11168 61 KEALFALEQKKLHNLLFCLADFKHKEELSSFREVMHTSKKGAKRLFEADFEEKIEILKTF
Ca1352 61 KEALFALEQKKLHNLLFCLADFKHKEELSSFREVMHTSKKGAKRLFEADFEEKIEILKTF
Ca2426 61 KEALFALEQKKLHNLLFCLADFKHKEELSSFREVMHTSKKGAKRLFEADFEEKIEILKTF
Ca0121 61 KEALFALEQKKLHNLLLCLADLKYKEELSSFREVMHTSKKEAKRLFEADFEEKIEILKTF

NCTC11168 121 NIPFEACIKNEELGFLEKFLSKNPNLKVVLNHLGSPKINRLNEYKKDLSFLKKFQNLYIK
Ca1352 121 NIPFEACMKNEELSFLENFLNKNSNLKVVLNHLGSPKINRLNEYKKDLNLLKKFPNLYIK
Ca2426 121 NIPFEACMKNEELSFLENFLNKNSNLKVVLNHLGSPKINRLNEYKKDLNLLKKFPNLYIK
Ca0121 121 NIPFEACMKNEELSFLEKFLNKNPNLKVVLNHLGSPKIDRLNEYKKDLNLLKKFPNLYIK

NCTC11168 181 LSIPDGFSQETPKEFIFELFAFLKENFSENKFIFGSNYPVAKITPAKWAKLIIESKIFDD
Ca1352 181 LSIPDGFSQETPKEFIFELFAFLKENFSENKFIFGSNYPVSNLSPKQWIELIMQSKVFKD
Ca2426 181 LSIPDGFSQETPKEFIFELFAFLKENFSENKFIFGSNYPVSNLSPKQWIELIMQSKVFKD
Ca0121 181 LSVPDDFSEQTSKEFIYELFAFFKENFSEDKFIFGSNYPVAKIAPAKWAKLIIESKIFKN

NCTC11168 241 LDKIFYKNALLIYKGG
Ca1352 241 LDLIFYKNALSIYKGE
Ca2426 241 LDLIFYKNALSIYKGE
Ca0121 241 LNKIFYQNALSIYKED

***Cj0488***

NCTC11168 1 MQRYGQIIKIKKEKIEEYKELHTKPYEGVCEMIKECNIQNYSIYLFGEYLFAYFEYIGAD
Ca1352 1 MQRYGQIIKIKKEKIQKYKNLHAKPYEGVCEMIKECNIQNYSIYLFGEYLFAYFEYVGAD
Ca2426 1 MQRYGQIIKIKKEKIQKYKNLHAKPYEGVCEMIKECNIQNYSIYLFGEYLFAYFEYVGAD
Ca0121 1 MQRYGQIIKIKKEKIEEYKNLHAKPYEGVCEMIKKCNMQNYSIYLFGEYLFAYFEYVGAD

NCTC11168 61 FEADMAKMARDESTQKWWKVTDPCQISLGYAGQKWLNMEEVFHLD
Ca1352 61 FEADMAKMARDENTQKWWKVTDPCQISLGYAGQKWLNMEEVFHLD
Ca2426 61 FEADMAKMARDENTQKWWKVTDPCQISLGYAGQKWLNMEEVFHLD
Ca0121 61 FEADMAKMARDESTQKWWKVTDPCQISLGYAGQKWLNMEEVFHLD

***Cj0489 + Cj0490***

NCTC11168 1 MTTYLNYIDGKFIPHNGEFIEVLNPATKEVISRVASASLEDTKRAIEAAKKAQKVWEAKP
Ca1352 1 MTTYLNYIDGEFIPHNGDFIEVLNPATKEVISKVASASLEDAKRAIEAAKKAQKSWETKS
Ca2426 1 MTTYLNYIDGEFIPHNGDFIEVLNPATKEVISKVASASLEDAKRAIEAAKKAQKSWETKS
Ca0121 1 MTTYLNYIDGKFIPHNGEFIEVLNPATKEVISRVASASLEDTKRAIEAAKKAQKVWEAKP


NCTC11168 61 AIERANHLKEIAS*YAKMLIS**PKFMQEQGKTRVLASIEINFTADYMDYTAEWARRYEG
Ca1352 61 AIERANHLREIASLIRKNANFLTEILMQEQGKTRALASVEVNFTADYMDYTAEWARRYEG
Ca2426 61 AIERANHLREIASLIRKNANFLTEILMQEQGKTRALASVEVNFTADYMDYTAEWARRYEG
Ca0121 61 AIERANHLKEIASLIRKNANFLTEVLMQEQGKTRVLASIEINFTADYMDYTAEWARRYEG


NCTC11168 118 EIIQSDRANEHIYLYKSAIGVIGGILPWNFPFFLIARKMAPALLTGNTIVIKPSSETPNN
Ca1352 121 EIIQSDRANEHIYLYKSAIGVIGGILPWNFPFFLIARKMAPALLTGNTIVIKPSSETPNN
Ca2426 121 EIIQSDRANEHIYLYKSAIGVIGGILPWNFPFFLIARKMAPALLTGNTIVIKPSSETPNN
Ca0121 121 EIIQSDRANEHIYLYKSAIGVIGGILPWNFPFFLIARKMAPALLTGNTIVIKPSSETPNN


NCTC11168 178 AFEFAKLVSQSSLPKGVFNLVAGKGSVVGYELSSNENIGMVSLTGSVEAGTRVMEAAAKN
Ca1352 181 AFEFVKLVSQSSLPKGVFNLVAGKGSVVGYELSSNENIGMVSLTGSVEAGARVMEAAAKN
Ca2426 181 AFEFVKLVSQSSLPKGVFNLVAGKGSVVGYELSSNENIGMVSLTGSVEAGARVMEAAAKN
Ca0121 181 AFEFAKLVSQSSLPKGVFNLVAGKGSVVGYELSSNENIGMVSLTGSVEAGTRVMEAAAKN


NCTC11168 238 IIKVSLELGGKAPAIVCKDADIDLAVEAIKASRICNNGQVCNCAERAYVHTSVYDEFVDK
Ca1352 241 IIKVSLELGGKAPAIVCKDADIDLAVEAIKASRICNNGQVCNCAERAYVHTSIYDEFVDK
Ca2426 241 IIKVSLELGGKAPAIVCKDADIDLAVEAIKASRICNNGQVCNCAERAYVHTSIYDEFVDK
Ca0121 241 IIKVSLELGGKAPAIVCKDADIDLAVEAIKASRICNNGQVCNCAERAYVHTSIYDEFVDK


NCTC11168 298 FVKAMSKVSVGNTLKGDFDMGPLVNQAGVDNALAMLQRATAKGAIVECGGKITDTSGYYF
Ca1352 301 FVKAMSKVSVGNTLKGDFDMGPLVNQAGVDNALAMLQRATAKGAVVECGGKITDTSGYYF
Ca2426 301 FVKAMSKVSVGNTLKGDFDMGPLVNQAGVDNALAMLQRATAKGAVVECGGKITDTSGYYF
Ca0121 301 FVKAMSKVSVGNTLKGDFDMGPLVNQAGVDNALAMLQRATAKGAIVECGGKITDTSGYYF


NCTC11168 358 PASVLTNVKHEDEIMQKEIFAPILPIAKFDTLDEVIDMANDCEYGLTSSIYTQNLDIAMR
Ca1352 361 PASVLTNVKHEDEIMQKEIFAPILPIAKFDTLDEAIDMANDCEYGLTSSIYTQNLDIAMR
Ca2426 361 PASVLTNVKHEDEIMQKEIFAPILPIAKFDTLDEAIDMANDCEYGLTSSIYTQNLDIAMR
Ca0121 361 PASVLTNVKHEDEIMQKEIFAPILPIAKFDTLDEAIDMANDCEYGLTSSIYTQNLDIAMR


NCTC11168 418 ASREIKFGETYINRENFEAMQGFHAGFRKSGIGGADGKHGLEEYLATHVVYLQYNTNKQ*
Ca1352 421 ASREIKFGETYINRENFEAMQGFHAGFRKSGIGGADGKHGLEEYLATHVVYLQYNTNKQ*
Ca2426 421 ASREIKFGETYINRENFEAMQGFHAGFRKSGIGGADGKHGLEEYLATHVVYLQYNTNKQ*
Ca0121 421 ASREIKFGETYINRENFEAMQGFHAGFRKSGIGGADGKHGLEEYLATHVVYLQYNTNKQ*
